# Optogenetic induction of the schizophrenia-related endophenotype of ventral hippocampal hyperactivity causes rodent correlates of positive and cognitive symptoms

**DOI:** 10.1038/s41598-018-31163-5

**Published:** 2018-08-27

**Authors:** Amy R. Wolff, Alexei M. Bygrave, David J. Sanderson, Edward S. Boyden, David M. Bannerman, Dimitri M. Kullmann, Dennis Kätzel

**Affiliations:** 1 Department of Experimental Psychology, University of Oxford, Oxford, UK; 2 Institute of Neurology, University College London, London, UK; 3 Department of Psychology, Durham University, Durham, UK; 4 Media Lab and McGovern Institute, Massachusetts Institute of Technology, Cambridge, USA; 5 Institute of Applied Physiology, Ulm University, Ulm, Germany

## Abstract

Pathological over-activity of the CA1 subfield of the human anterior hippocampus has been identified as a potential predictive marker for transition from a prodromal state to overt schizophrenia. Psychosis, in turn, is associated with elevated activity in the anterior subiculum, the hippocampal output stage directly activated by CA1. Over-activity in these subfields may represent a useful endophenotype to guide translationally predictive preclinical models. To recreate this endophenotype and study its causal relation to deficits in the positive and cognitive symptom domains, we optogenetically activated excitatory neurons of the ventral hippocampus (vHPC; analogous to the human anterior hippocampus), targeting the ventral subiculum. Consistent with previous studies, we found that vHPC over-activity evokes hyperlocomotion, a rodent correlate of positive symptoms. vHPC activation also impaired performance on the spatial novelty preference (SNP) test of short-term memory, regardless of whether stimulation was applied during the encoding or retrieval stage of the task. Increasing dopamine transmission with amphetamine produced hyperlocomotion, but was not associated with SNP impairments. This suggests that short-term memory impairments resulting from hippocampal over-activity likely arise independently of a hyperdopaminergic state, a finding that is consistent with the pharmaco-resistance of cognitive symptoms in patients.

## Introduction

The pathophysiology of schizophrenia is complex, involving a wide range of brain areas and neurotransmitter systems. The hippocampus is implicated as having a central role, since it displays a number of alterations in schizophrenia, including reduced volume, changes in organisation and cytoarchitecture^1–4^, and altered expression of synaptic proteins^2–5^, and altered synaptic transmission^6–9^. Hippocampal dysfunction is also evident in imaging data from patients with schizophrenia, who show over-activation of the hippocampus, both at rest^10–13^, and during tasks requiring minimal cognitive load^14,15^. Notably, such over-activity of anterior CA1 and subiculum has been correlated with the severity of positive, negative and, more recently, cognitive symptoms of schizophrenia^10,11,16^. It is not clear, however, if these symptoms are directly *caused* by over-activity of hippocampal output.

Altered hippocampal structure and function, including reduced hippocampal volume^17^ and anterior hippocampal over-activity^11^ have also been reported in individuals at high-risk of developing schizophrenia. Evidence that hippocampal over-activity spreads from the CA1 region to the subiculum during the transition from the prodromal to the psychotic state^16^ suggests that altered hippocampal function reflects a primary stage of impairment in schizophrenia, and that therapeutic intervention to regulate hippocampal activity could be effective in preventing disease progression^16,18,19^. Hippocampal over-activity may therefore represent a crucial biomarker^20^, and elucidating the mechanisms whereby this activity contributes to the different symptom domains may be a critical step in drug discovery.

The rodent hippocampal formation, including the ventral hippocampus (vHPC; analogous to the human anterior hippocampus), and particularly the ventral subiculum (vSUB), can modulate the activity of dopaminergic neurons in the ventral tegmental area (VTA)^21–25^, and increase dopamine release in the nucleus accumbens (NAc)^26–30^. These changes to the midbrain dopaminergic system are also associated with hyperlocomotion^31,32^, a putative rodent correlate of positive symptoms in rodents^33^. It has therefore been proposed that hippocampal over-activity contributes to the hyperdopaminergic state underlying the emergence of positive symptoms of schizophrenia^34,35^. In support of this hypothesis, activation of the vHPC (with electrical or chemical stimulation) has previously been shown to produce rodent correlates of positive symptoms in translational paradigms such as pre-pulse inhibition and latent inhibition^36,37^. It has also been shown, in a developmental rat model of schizophrenia (methylazoxymethanol acetate), that the altered activity of VTA dopaminergic neurons and increased locomotor responses to amphetamine can be normalized by pharmacological inhibition of the vHPC^38^.

Genetically and spatially targeted manipulation of neural circuits using optogenetics provides a novel class of rodent models of psychiatric diseases, which are more specific and mechanistically defined than previous pharmacological or transgenic models^39^. With this approach, physiological endophenotypes (i.e. abnormal circuit activity) observed in patients can be precisely recreated in rodents, and can be used to test whether such abnormal physiological activity plays a causal role in specific symptoms. If so, they represent a biomarker in preclinical drug screening^39^ and clinical monitoring of therapeutic efficacy. Furthermore, this approach allows temporal control not afforded by previous techniques. For example, in contrast to chemical stimulation, optogenetics can model over-activation during specific epochs of behavioural tasks. Additionally, unlike electrical stimulation, manipulations can be targeted to the soma of specific cell types, leaving fibres of passage unaffected. Using such precise tools of control, the targeted re-creation of endophenotypes of abnormal neurophysiological activity observed in patients might be a promising novel route to derive optogenetic rodent models with relevance to psychiatric disorders^40^. This approach has, however, not yet been widely adopted in the context of schizophrenia^41–44^.

To explore the mechanistic link between increased hippocampal output, dopaminergic regulation, and cognitive impairment, we aimed to optogenetically re-create the endophenotype of anterior hippocampal over-activity observed in patients. The effects of electrical stimulation and pharmacological activation of the hippocampus in rodents are thought to be mediated by increased activity of excitatory projection neurons at the vHPC output stage – the vSUB^23–25,35^. Therefore, we aimed to selectively activate excitatory neurons of the vSUB, and assess the effects of this manipulation on novelty-induced hyperlocomotion and hippocampus-dependent short-term memory performance (assessed with the Y-maze spatial novelty preference task^45^). While the mechanisms whereby increased hippocampal activity leads to hyperlocomotion in rodents have been extensively studied^29,46,47^, the link between hippocampal over-activity and cognitive impairment are less clear^48^.

## Results

### Histology

**Figure 1:**
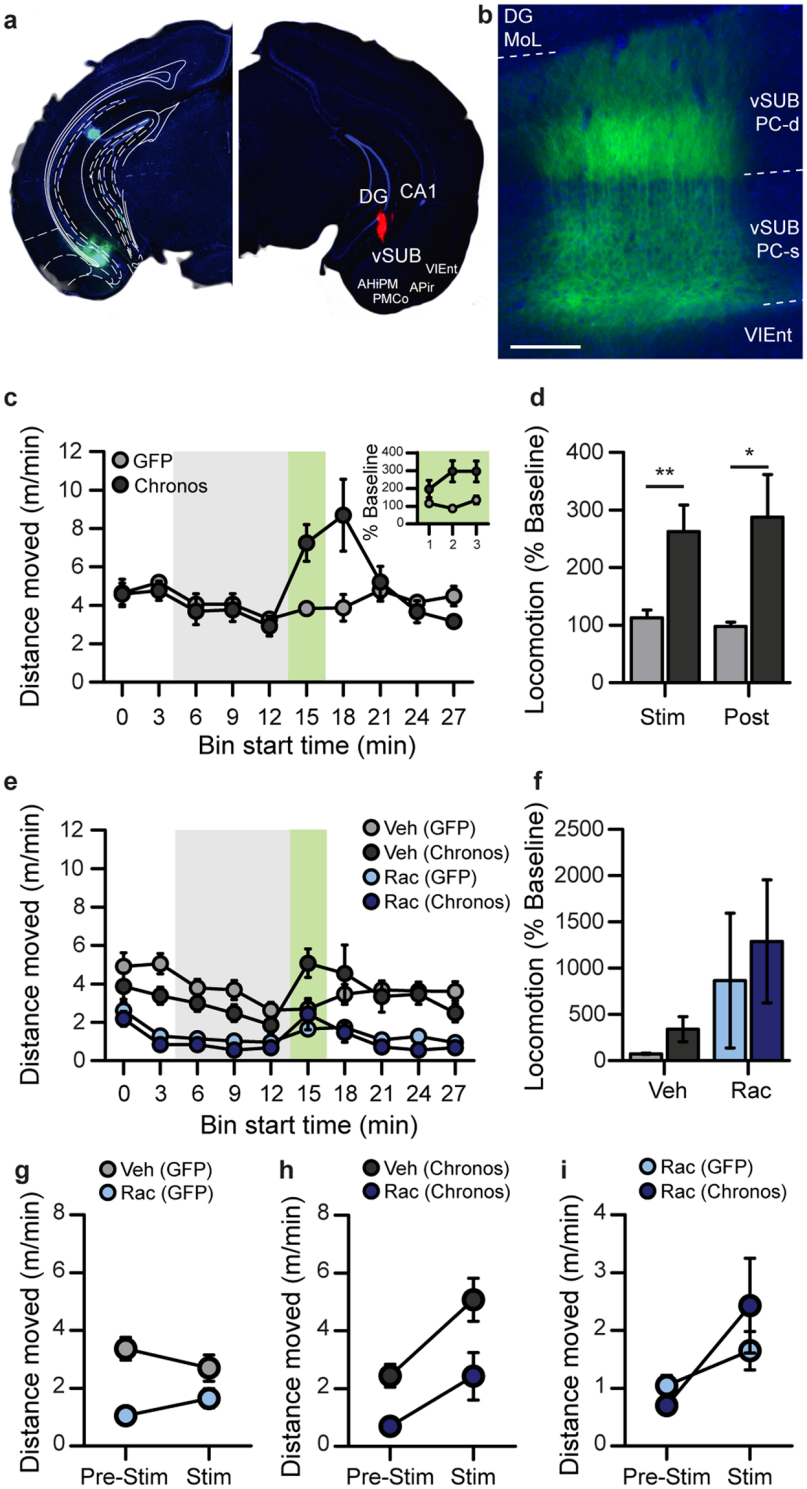
Optogenetic activation of the vHPC induces hyperlocomotion. (**a**) Selective transfection of the vHPC (vSUB & vCA1) is indicated by GFP-fluorescence (left) derived from the transfected Chronos-GFP expression cassette. Blue background stain with DAPI. Note that the dorsal fluorescence is autofluorescence from damage to the tissue, rather than Chronos-GFP expression. Photomicrograph of the location of vertically implanted optic fibre coated in DiI (red) 100–200 µm above the pyramidal cell layer of the vHPC (right; DiI was not applied in experimental animals). (**b**) High-resolution image to illustrate the membrane-bound expression of Chronos-GFP (green) in the somata (vSUB PC-s) and dendrites (vSUB PC-d) of pyramidal cells of the vSUB. Scale bar = 100 µm; blue, DAPI stain; the ventral pole is left, the dorsal pole right. This image is from a separate animal not used for behavioural testing. (**c**) Optogenetic induction of hyperlocomotion during stimulation (block starting at minute 15; green shaded area) in the pre-screened cohort in a novel open field. Inset (top right) shows locomotion calculated for each min of the 3 min stimulation as a % of baseline (min 15–18). (**d**) Data of (**c**) expressed as distance moved during the stimulation-phase (during-stim; green shading), and the first post-stimulation block (post-stim) as a percentage of the average distance moved during baseline (3 blocks immediately before stimulation; grey shading). (**e**,**f**) Same experiment as in (**c**), with injection of vehicle (Veh; grey) or the D2R antagonist raclopride (Rac; 1 mg/kg; blue) 1 h before the start of the experiment. Optogenetic stimulation in minutes 15–18 (green shading), with baseline period shown in grey shading. (**f**) Locomotion during optogenetic stimulation of the vHPC (stimulation as % of baseline) following vehicle (grey) or Raclopride (blue) injection. (**g–i**) Absolute changes in locomotion from baseline to stimulation phase are shown separately for the GFP controls (**g**) and Chronos animals (**h**), and for both groups following Raclopride injection (**i**). Indication of significant effects or interactions are omitted from (**c**, **e**, **f**, and **g**–**i**) for clarity, see main text for analysis. Data are shown as mean ± SEM; *p < 0.05, **p < 0.01, Kolmogorov-Smirnov Z Test. Abbreviations, DG: dentate gyrus; DG MoL, molecular layer of the DG; CA1: cornu ammonis area 1; vSUB: ventral subiculum; APir: amygdalopiriform transition area; VIEnt: ventral intermediate entorhinal cortex; AHiPM: amygdalohippocampal area, posteromedial part; PMCo: posteromedial cortical amygdaloid area.

To allow activation of excitatory vSUB output neurons, we injected the optogenetic activator Chronos bilaterally into the vHPC of CamKIIα-*Cre* mice, and implanted optic fibres for light delivery. Optic fibre tracts were not visible in their entirety in the sliced tissue; therefore the precise location of the tips of the optic fibres could not be determined in every experimental animal. Additional animals in which optic fibres were coated in fluorescent DiI and implanted at the same coordinates demonstrated that the placements effectively targeted light towards the vSUB (see Fig. 1a). Furthermore, in cases where partial tracts were visible in experimental animals, the AP coordinates and ML locations were entirely consistent with these data.

All Chronos-transfected animals meeting the optogenetic stimulation calibration criteria (see Methods; *n* = 18) had expression in the targeted vSUB area in the stimulated hemisphere (Fig. 1a; left panel, Fig. 1b). Many animals also had some strong expression in parts of the adjacent ventral CA1 subfield (16/18) and/or weak expression in the ventral DG (14/18) and CA3 (9/18). Viral expression outside the vHPC was seen in only 4/20 animals, where weak expression was also evident in parts of the amygdalopiriform transition area, posteromedial cortical amygdaloid area, and the posteromedial amygdalohippocampal area, which are adjacent to the vSUB. Given the placement of the optical fibre just above vSUB (see Fig. 1a; right panel), the low light powers used, and weak offsite expression, it is unlikely that the light effectively stimulated transfected subfields outside of the vSUB/vCA1. Nevertheless, we refer to our manipulation as vHPC (not vSUB) stimulation, to acknowledge the potential contribution of induced overactivity in adjacent hippocampal subfields to the observed behavioural effects.

### Behaviour

#### Calibration of optical stimulation intensity

We first calibrated stimulation levels in the Chronos group to determine the level of stimulation just sufficient to produce hyperlocomotion via either one of the bilaterally implanted optic fibres (i.e. hemispheres, see Methods). The individually determined power values (mean power = 3.4 mW, range: 2–5 mW) and the unilateral stimulation configuration were kept for the remainder of the test battery to minimize the risk of optogenetic over-stimulation or the induction of seizures. Animals exhibiting seizures (*n* = 4), or failing to show hyper-locomotion with optical stimulation at 5 mW in either hemisphere (*n* = 7) during calibration were excluded from all subsequent tests. Our observations indicated that susceptibility to seizures was likely due to strong off-target expression in the dentate gyrus and/or CA3 seen in some mice.

#### Optogenetic stimulation of the vHPC induces hyperlocomotion

After calibration, we confirmed that the same stimulation intensity elicited hyperlocomotion in a novel open field. Locomotor activity (distance travelled) was measured for 30 minutes and scored in 3-min bins, with 3 minutes of stimulation starting at minute 15. We calculated the distance travelled during the stimulation block as a percentage of the average distance travelled during the baseline period (previous 3 bins; 3 min each; from min 6) to assess hyperlocomotion. Again, we found that the change in locomotion during optogenetic stimulation in Chronos-transfected animals was significantly higher than in GFP controls (Fig. 1c,d; stimulation as % of baseline: D(28) = 1.59, *p* = 0.01; Kolmogorov-Smirnov Z Test; *n* = 14 Chronos, 16 Controls). The relative increase in locomotion during the stimulation period was significantly greater than baseline (100%) in Chronos animals (*p* = 0.004; One-Sample Wilcoxon Sign Rank Test), but not in GFP-transfected controls (*p* = 0.92; One-Sample Wilcoxon Sign Rank Test). The effects of stimulation on locomotion showed a non-significant trend to increase over each minute of the 3 min stimulation in Chronos animals (see Fig. 1c, inset; χ^2^(2) = 2.71, *p* = 0.26; Related-Samples Friedman’s). Additionally, we found that locomotion relative to baseline was significantly higher in the Chronos group than in controls during the 3 min block *after* stimulation (Fig. 1d; minutes 18–21; first post-stim block as % of baseline: D(28) = 1.39, *p* = 0.04, Kolmogorov-Smirnov Z Test). As unilateral manipulations may produce turning behaviour^49^, we also assessed rotations during the optogenetic stimulation. Chronos animals turned significantly more than GFP-transfected controls during the stimulation (3 min; t (28) = 2.32, *p* = 0.03; number of turns: Chronos = 8.4 ± 1.1, GFP = 5.6 ± 0.6; Mean ± SEM). However, there were no group differences in the percentage of contralateral turns during stimulation (% controlateral rotations; D(28) = 0.66, *p* = 0.78, Kolmogorov-Smirnov Z Test), and there was no evidence of bias for turning towards the contralateral side (% contralateral turns: Chronos = 49.7 ± 6.8%, GFP = 56.3 ± 6.6%; Mean ± SEM). It is likely that increased rotations in the Chronos group reflect the general increase in locomotor activity resulting from vHPC stimulation, rather than an induction of circling.

#### Role of dopamine D2-receptors in mediating hyperlocomotion induced by optogenetic stimulation of vHPC

According to a prominent circuit model of schizophrenia^21^ the primary, albeit indirect, downstream effect of an over-active vHPC is increased burst firing of dopaminergic neurons in the VTA, which innervate the NAc. This leads to the prediction that the transient increase in locomotion caused by optogenetic stimulation of the vHPC (Fig. 1c,d) should be eliminated by D2R-antagonism. To test this hypothesis, we injected raclopride, a selective and potent D2/3-antagonist (Rac; 1 mg/kg), or vehicle (Veh) one hour before testing the effect of optogenetic vSUB stimulation (Fig. 1e,f). We observed a strong suppression of baseline locomotion by raclopride, which confounds further analysis of *relative* locomotor activity during stimulation normalized to baseline locomotion (see Fig. 1f for relative values calculated as for Experiment 1).

Instead, we assessed the *absolute* values of locomotion using a 3-way ANOVA with repeated measures of phase (baseline vs. stimulation) and drug (vehicle vs. raclopride), and a between-subject factor of group (Chronos vs. GFP). Relative to vehicle, raclopride strongly suppressed locomotion in both groups throughout testing, as evidenced by a main effect of drug and the lack of significant drug × group and drug × phase interactions (see Fig. 1e; drug: F(1, 26) = 51.58, *p* < 0.001; drug × group: F(1, 26) = 0.90, *p* = 0.35; drug × phase: F(1, 26) = 0.24, *p* = 0.63). There was no overall effect of group (F(1, 26) = 1.08, *p* = 0.31), but a significant main effect of phase (F(1, 26) = 9.86, *p* = 0.004), indicating a locomotor-increasing effect of the optical stimulation irrespective of drug and group (see Fig. 1e–h). Furthermore, the group × phase interaction (F(1, 26) = 10.46, *p* = 0.003), and group × phase × drug interactions were both significant (F(1, 26) = 8.63, *p* = 0.007).

To further explore these interactions, separate 2-way repeated-measures ANOVAs analysing the effect of drug and test phase were conducted within each group (Fig. 1g,h). In the Chronos group, there was a significant effect of phase and drug (phase: F(1, 11) = 8.03, *p* = 0.02; drug: F(1, 11) = 41.16, *p* < 0.001), but no significant drug × phase interaction (F(1, 11) = 1.61, *p* = 0.23), implying that raclopride reduced baseline locomotion throughout, but was ineffective against the laser-induced increase of locomotion (Fig. 1h). In contrast, in the GFP controls, there was a significant drug × phase interaction (F(1, 15) = 12.73, *p* = 0.003), but, in turn, no main effect of phase (F(1, 15) = 0.05, *p* = 0.83), while a significant effect of drug (F(1, 15) = 18.60, *p* = 0.001) remained. This implies (see Fig. 1g) that, although the stimulation did not increase locomotion in the GFP-group *in general*, there was a stimulation-induced increase exclusively in the raclopride condition. This may be due to reinvigoration of exploration by the presentation of a prominent visual cue (the laser light) on a low background level of locomotion. An additional 2-way group × phase ANOVA within the raclopride condition revealed a significant effect of phase (F(1, 26) = 7.98, *p* = 0.009), but no main effect of group (F(1, 26) = 0.26, *p* = 0.62), or group × phase interaction (F(1, 26) = 1.87, *p* = 0.18). This indicates that the stimulation-induced increase in locomotion in the raclopride-treated Chronos group was not significantly greater than the stimulation-related increase seen in raclopride-treated GFP controls (Fig. 1i). Together, these results indicate that raclopride failed to prevent the specific increase of locomotion during the stimulation period, despite effective suppression of baseline locomotion. However, as there was no significant group difference under raclopride, it is possible that this raclopride-resistant locomotor increase reflects non-specific arousal caused by the laser pulses, rather than a direct optogenetic effect.

#### Optogenetic stimulation of vHPC alters spatial novelty preference

**Figure 2:**
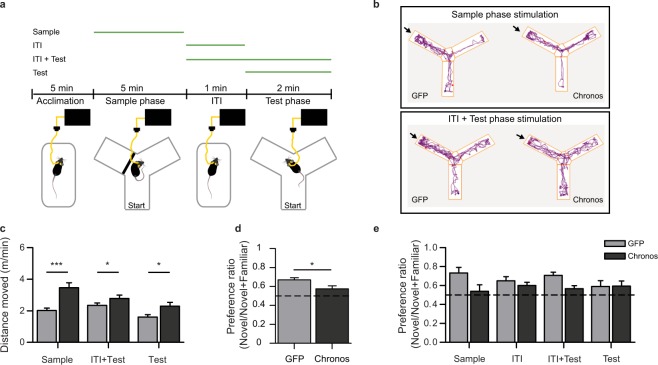
Optogenetic impairment of spatial novelty preference (SNP). (**a**) Illustration of the Y-maze test protocol used to assess SNP. In all cases animals were familiarized to a novel cage and the tether before introduction into the Y-maze (acclimation). Green lines above indicate the phase of optogenetic stimulation for the 4 different stimulation phases used: (1) sample phase (Sample) (2) Intra-trial interval (ITI) (3) test phase (Test) and (4) ITI and test phase (ITI + Test). (**b**) Example path lengths from individual GFP (left) and Chronos animals (right) are shown for exploration during the test phase following stimulation in the sample phase (top) or the ITI + Test phase (bottom). The novel arm is indicated by the arrow. (**c**) Optogenetically-induced hyperlocomotion was observed during stimulation for each stimulation phase during which locomotion was recorded; locomotion was not tracked in the holding cage, and is therefore not shown for the ITI only stimulation condition, and data for ITI + Test stimulation are shown for the test phase only. (**d**,**e**) Preference ratios for the novel arm calculated as the ratio of the time spent in the novel goal arm to the time spent in both goal arms. Data are presented averaged across all 4 stimulation phases (reflecting the main effect of group from the ANOVA; **d**) or individually for each stimulation protocol (**e**). The dashed lines (**d**–**e**) indicate chance level (0.5), i.e. equal preference for both goal arms. Data are shown as mean ± SEM; *p < 0.05, ***p < 0.001; *t*-Test or Kolmogorov-Smirnov Z-Test.

We next used a task assessing hippocampus-dependent short-term memory^45^ to investigate the effect of vHPC over-activity on cognitive function. We tested spatial novelty preference in the Y-maze following optogenetic activation of the vHPC during different phases of the task (see Fig. 2a,b). We stimulated the vHPC either during the sample phase (5 min), the ITI (1 min), the test phase (2 min), or throughout both the ITI and the subsequent test phase (3 min). Novelty-preference was calculated as the ratio of time spent in the novel arm divided by time spent in both choice arms combined (excluding the start arm). Locomotion was assessed by distance travelled (m) and was converted to speed (m/min) to normalize data relative to the duration of each phase of testing.

We verified that Chronos animals (*n* = 10) showed hyperlocomotion relative to GFP controls (*n* = 16) during the phase of the test in which stimulation was applied (Fig. 2c; Sample: t(24) = 4.54, *p* < 0.001; Test: D(24) = 1.43, *p* = 0.034; ITI + Test: D(24) = 1.36, *p* = 0.048; activity levels were not tracked in the holding cage and therefore locomotor data for the ITI-only condition are not available). This hyperactivity confirmed that optogenetic stimulation was effective.

Optogenetic vHPC stimulation did not affect the time spent in both goal arms combined during the test phase (NOV + FAM; Sample: t(24) = −0.43, *p* = 0.67; ITI: t(24) = 0.52, *p* = 0.61; Test: D(24) 0.78, *p* = 0.59; ITI + Test: t(24) = 0.679, *p* = 0.50). However, vHPC stimulation significantly impaired spatial novelty preference when conducting an ANOVA with stimulation condition (ITI + Test, ITI, Test, Sample) as a repeated measure in those animals where data was available for all stimulation conditions (Fig. 2d; *n* = 10 Chronos, 16 GFP). There was a significant main effect of group (GFP vs Chronos; F(1, 24) = 6.32, *p* = 0.019), but no significant effect of stimulation condition (F(3, 72) = 0.31, *p* = 0.819), and no interaction (F(3, 72) = 1.36, *p* = 0.262), indicating that stimulation of the vHPC during any stage of testing in Chronos animals impaired task performance to some degree (Fig. 2b,d,e). Importantly, SNP impairments were not due to insufficient exploration during the sample phase, as the time spent in the open goal arm during the sample phase did not differ between groups for any of the stimulation paradigms (Sample: t(24) = −1.38, *p* = 0.18; ITI + Test: t(24) = 1.02, *p* = 0.32; ITI: D(24) = 0.37, *p* = 1.0; Test: t(24) = −0.2, *p* = 0.85).

#### Optogenetic stimulation of the vHPC does not alter anxiety or induce place preference

Optogenetic induction of vHPC over-activity impaired SNP, implying an impairment of short-term memory. However, novelty-preference could also be altered due to emotional or motivational changes, rather than cognitive deficits. Firstly, activation of the vHPC could reduce the preference for novel places by altering the balance between exploratory drive and unconditioned anxiety^23^, since manipulations of the ventral hippocampus may affect anxiety^50–53^. If vHPC activation increases anxiety, this could compete with the drive towards exploration of the novel arm during the novelty preference test. A second potential confound is that the optogenetic stimulation itself, through downstream effects on the dopaminergic system^54^, might be perceived as rewarding, and therefore induce a place preference (PP) for the location in which the optogenetic stimulation occurred (i.e. the familiar arm when stimulated during the sample trial). This preference would then conflict with the drive to explore the novel arm for the sample-only stimulation condition.

**Figure 3:**
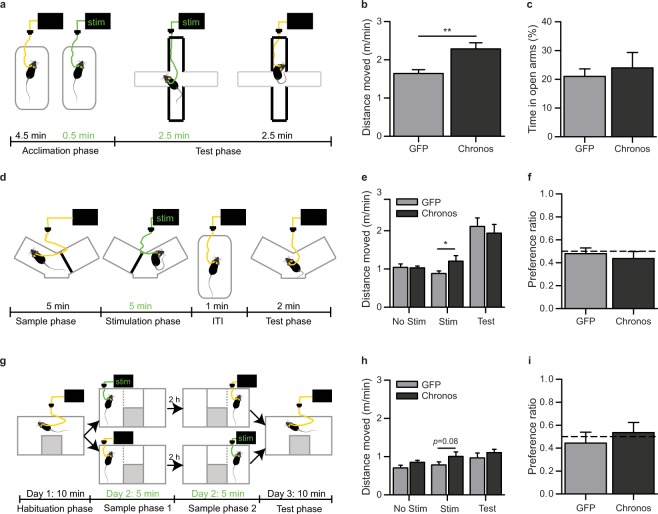
Optogenetic activation of the vHPC does not alter anxiety or place preference. (**a**) Experimental paradigm used to assess anxiety following optogenetic stimulation (green), with bold lines indicating walled arms of the elevated plus-maze (EPM) apparatus. Locomotion (**b**) and preference for the open arm (**c**) on the EPM. Data are presented for the first half (2.5 min) of the test on the EPM (when optogenetic stimulation was applied). Preference for the open arms was measured as the percentage of time spent in the open arms relative to time spent in all open and closed arms. (**d**) Experimental paradigm for testing short-term place preference (PP) in the modified Y-Maze apparatus, delivery of optogenetic stimulation is shown in green. (**e**) Locomotor activity in the individual phases of the test: first sample phase, with no stimulation (No Stim); second sample phase, with stimulation (Stim); and test phase (Test). (**f**) Preference ratio measured as time spent in the goal arm of the Y-maze in which optogenetic stimulation was delivered relative to time in both goal arms. Dashed line indicates chance performance (i.e. equal preference for both goal arms). (**g**) Experimental paradigm for testing long-term place preference in a modified 3-chamber apparatus. (**h**) Locomotion during different phases of test. (**i**) Preference ratios for time spent in the chamber of the apparatus in which stimulation was applied during the test phase conducted 24 h later. Dashed line indicates chance performance (i.e. equal preference for both goal arms). *p < 0.05, **p < 0.01; *t*-Test or Kolmogorov-Smirnov Z-Test. Data are shown as mean ± SEM.

We assessed both these potential explanations. First, we used the elevated plus- maze to measure exploration/anxiety balance and stimulated the vHPC starting 30 s before placement on the maze, and continuing for the first half (2.5 min) of maze exploration (see Fig. 3a). Again, we successfully induced hyperlocomotion compared to GFP-controls during the stimulation period (Fig. 3b; t(25) = 3.45, *p* = 0.002; *n* = 13 Chronos, 14 GFP). However, the preference for the time spent in the open arms during stimulation was not altered in the Chronos group compared to controls (Fig. 3c; D(25) = 0.49, *p* = 0.973; Kolmogorov-Smirnov Z Test).

Second, to test for the possibility of an optogenetic induction of a place preference using the same exposure (5 min) and ITI times (1 min) as the Y-Maze task, we adapted this paradigm to resemble a conditioned place preference paradigm (see Fig. 3d). In contrast to the SNP test, mice sampled *both* goal arms separately before the test phase, while only exploration of the second goal arm was accompanied by optogenetic stimulation of the vHPC. Again, we could induce hyperlocomotion in Chronos animals selectively during the optogenetic stimulation phase compared to controls (Fig. 3e; Non-Stim: t(27) = −0.10, *p* = 0.92: *n* = 15 Chronos, 14 GFP; Stim: t(25) = 2.08, *p* = 0.048: *n* = 13 Chronos, 14 GFP; Test: t(27) = −0.55, *p* = 0.59: *n* = 15 Chronos, 14 GFP). Nevertheless, there was no evidence of a subsequent preference for the arm that was paired with stimulation in Chronos animals (preference vs 0.5; t(14) = −1.03, *p* = 0.32), and no significant difference between the groups (Fig. 3f; t (1, 27) = −0.52*, p* = 0.606; *n* = 15 Chronos, 14 GFP).

Note, however, that in this paradigm, it was not possible to counterbalance the order of stimulation vs no stimulation due to potential carry-over effects of optogenetic stimulation. (i.e. Stimulation during the first sample would also potentially effect the second sample), as hyperlocomotion persisted for at least 3 min after stimulation; (see Fig. 1b,c). Counter-balancing the order of stimulation would have additionally separated the stimulation from the test phase by delays longer than those used in the Y-maze task.

Nevertheless, it is therefore possible that the lack of any effect results from competition between place preference (preferred exploration of the stimulated arm), and recency, which would drive preference for the non-stimulated arm (i.e. the less recently experienced arm). To address this issue, we conducted a long-term place preference task during which the sample phases were separated by 2 hours, allowing for counterbalancing of the stimulation/no stimulation order (see Fig. 3g). Note that Chronos animals only showed a trend towards hyperlocomotion during the stimulation in this case (Fig. 3h; D(28) = 1.27, *p* = 0.08; Kolmogorov-Smirnov Z Test). However, in the test phase (24 h later), there was still no evidence of a preference for the stimulated chamber of the apparatus in Chronos animals (preference ratio vs 0.5; t(14) = 0.420, *p* = 0.68), and no significant group difference (Fig. 3i; D(28) = 0.85, *p* = 0.459; Kolmogorov-Smirnov Z Test, *n* = 14 Chronos, 16 GFP). Together, these results indicate that optogenetic stimulation does not impair novelty preference by increasing anxiety or the rewarding value associated with the stimulated arm.

#### Increased dopamine transmission does not impair spatial novelty preference

**Figure 4:**
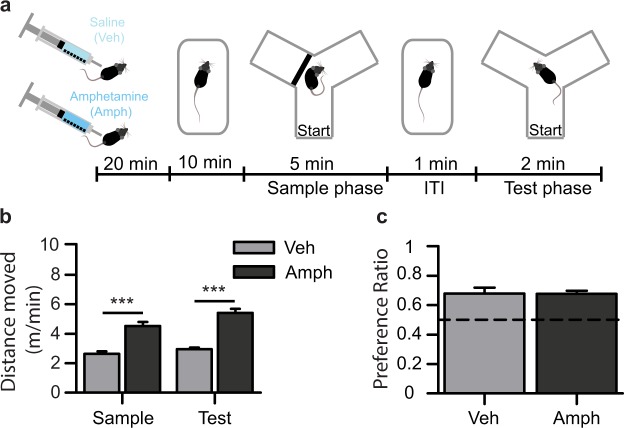
Increased dopamine is not sufficient to induce impairments in spatial novelty preference. (**a**) Experimental paradigm for assessing the effects of the dopamine agonist amphetamine (2.5 mg/kg; Amph) administered 30 min before testing on (**b**) hyperlocomotion and (**c**) novelty preference. (**b**) Amphetamine administration in wild type mice produced hyperlocomotion relative to vehicle-treated controls (Veh) during both the sample and test phases of the SNP task. (**c**) Novelty preference ratios for the time spent in the novel arm relative to the time spent in both goal arms combined did not differ between Amph and Veh injected mice, and performance in both groups was above chance level (Dashed line = 0.5; i.e. equal preference for both goal arms). ***p < 0.001, *t*-Test.

Given that optogenetic vHPC stimulation impaired spatial short-term memory during the SNP task, and that the hyperactivity induced by vHPC stimulation is associated with increased dopaminergic transmission^26,27,31^, we asked whether increased dopamine transmission alone is sufficient to alter SNP. We assessed SNP in control mice injected with the pro-dopaminergic drug amphetamine (Fig. 4a; Amph), at a dose known to produce robust locomotor hyperactivity^55^. As expected, amphetamine (2.5 mg/kg) led to strong hyperlocomotion relative to vehicle-injected mice (Veh) during both the sample and test phases of the task (Fig. 4b; sample: t (21) = −5.63, *p* < 0.001; test: t (13.79) = −8.62, *p* < 0.001). Amphetamine administration did not alter the time spent exploring the goal arm during the sample phase (t (21) = −1.43, *p* = 0.17), or the time spent in both goal arms combined during the test phase (Novel + Familiar; t (21) = 0.37, *p* = 0.71). Importantly, amphetamine-treated mice showed a similar novel arm preference to vehicle-injected controls (Fig. 4c; t(15.68) = 0.06, *p* = 0.95). Furthermore, preference ratios for both groups were significantly above chance (Veh: t(10) = 4.51, *p* = 0.001; Amph: t(11) = 8.17, *p* < 0.001), i.e. both groups spent significantly more time exploring the novel compared to the familiar arm, suggesting that they had intact short-term memory for the arm visited in the sample phase.

## Discussion

Here we aimed to model schizophrenia-related over-activity of ventral hippocampal (vHPC) output by using optogenetics to selectively activate excitatory projection neurons of the vHPC, specifically targeting the ventral subiculum (vSUB). In addition to high spatial, temporal and genetic resolution, allowing more controlled modelling of vHPC hyperactivity than earlier approaches (chemical/electrical stimulation), the calibration of stimulation power for each animal allows for a similar level of activation across animals. While the applied stimulation frequency of 20 Hz probably does not exactly recapitulate the hippocampal hyperactivity seen in patients and may not represent endogenously produced spike activity, it is known to effectively increase dopamine transmission in the nucleus accumbens (NAc) and locomotor activity in rodents when used for electrical vHPC stimulation^28–30^. It therefore effectively models the hypothesized hyperdopaminergic downstream effects of such changes in the anterior HPC in patients. We found that *optogenetic* induction of vHPC over-activity causes robust hyperlocomotion and a hippocampus-dependent memory deficit; putative rodent correlates of positive and cognitive symptoms of schizophrenia, respectively. We also show that these features are likely dissociable, as only the induction of hyperlocomotion is mimicked by elevating dopaminergic signalling.

The locomotion-promoting effect of the previously described pharmacological or electrical stimulation of the vHPC^22,29,47^ has been proposed to result from increased firing of VTA dopamine neurons, and subsequent increases in dopamine release in the NAc^21,26,38^. D2 receptors in the NAc are known to be necessary for mediating spontaneous locomotion^56^. To establish whether such a D2-dependent mechanism also underlies the hyperlocomotion produced by optogenetic stimulation of the vHPC, we tested the effect of the D2-antagonist raclopride on optogenetically-induced hyperlocomotion. Raclopride did not significantly decrease the effect of optogenetic stimulation on relative levels of locomotion, akin to hyperlocomotion produced by electrical vHPC stimulation in rats^29^. However, we also observed an increase in locomotion during stimulation in the GFP control group treated with raclopride, which might have resulted from the salient visual stimulus of the laser light re-invigorating exploration in these partially sedated animals. Consequently, the group comparison within the raclopride condition revealed statistically similar stimulation-induced increases in locomotion in both groups, raising the possibility that the raclopride-resistant increase in the Chronos group was induced non-specifically by the laser light rather than optogenetically.

We also found that optogenetic stimulation of the vHPC impaired performance on a test of hippocampus-dependent short-term memory, suggesting that hippocampal over-activity in rodents may lead to phenotypes that are potentially relevant to the cognitive symptoms of schizophrenia. Furthermore, these differences in SNP are not explained by alterations in anxiety or potential rewarding effects of vHPC optogenetic stimulation. A previous study found that *silencing* hippocampal projections to the medial prefrontal cortex (mPFC) specifically during the encoding, but not the retrieval phase of a spatial short-term memory task reduced performance^57^. In contrast, in our study, the lack of an interaction between group and stimulation phase indicated that *activation* of the vHPC is detrimental to spatial short-term memory regardless of whether stimulation occurs during the encoding or retrieval phases of the task. This could arise from disruptions of state-dependent memory processes, whereby performance is impaired as a result of changes in behavioural state between encoding and retrieval. However, given that the effects of optogenetic stimulation (and presumably the associated behavioural state) were found to outlast the stimulation period on a timescale of several minutes (see Fig. 1b,c), the impairment in animals stimulated during the sample phase only would argue against this explanation.

An important question relates to the mechanism underlying the cognitive impairment. In particular, we addressed whether the short-term memory impairment induced by over-activation of the vHPC could result from the well-described induction of a hyperdopaminergic state. It was not possible to evaluate the role of dopamine signalling in this study using the same pharmacological approach that was used previously in the hyperlocomotion test, as raclopride was found to decrease baseline locomotion in all animals, and exploration is critical for the novelty preference task. Therefore, we did the reverse experiment, and tested whether pharmacologically *increasing* dopamine signalling alone was sufficient to induce impairments in the Y-maze SNP task. We found that wild-type mice injected with a dose of amphetamine sufficient to produce robust hyperlocomotion did not show short-term memory impairments. This suggests that the cognitive impairment induced by vHPC hyperactivity is likely independent of increased dopamine transmission. Admittedly, this conclusion is indirect, as it is unclear if systemic amphetamine at the given dose reproduces the effect of optogenetic vHPC stimulation on dopamine neurotransmission in terms of magnitude, time course, and regional specificity. Nevertheless, the present results do show that an impairment in SNP cannot simply arise from an elevation of dopamine signalling, or locomotor hyperactivity alone.

This model of optogenetic over-activation provides a valuable tool to study which schizophrenia-related deficits and symptom domains may be caused by an overactive hippocampus, and to what extent elevated dopamine signalling is involved, or indeed, other downstream brain structures. Manipulations of the dorsal HPC, the nucleus reuniens of the thalamus, and the mPFC have been shown to alter performance on working memory tasks^44,58–60^, and the hyperactivity of the vHPC might influence short-term memory through direct projections via these regions, independent of polysynaptic loops through the dopamine system^61,62^. This would support our claim that the dopamine-related phenotypes and the memory impairments induced by vHPC stimulation are dissociable. For example, in a direct comparison of the effects of pharmacological or electrical stimulation of the dorsal vs. the ventral hippocampus, only the latter resulted in increased activity of the mesolimbic dopamine system and produced phenotypes linked to the positive symptom domain^37,63^. This anatomical separation of downstream effects of vHPC hyperactivity is consistent with the observation that antipsychotics, which act primarily on the dopamine system, are largely ineffective against cognitive symptoms of schizophrenia.

## Materials and Methods

### Surgery

To allow activation of excitatory vSUB output neurons, we exploited the fast kinetics and high apparent light sensitivity of the optogenetic activator Chronos (ChR90), with GFP as a reporter protein^64^. Twenty-nine adult male CamKIIα-*Cre* mice on a C57BL/6 background (B6.Cg-Tg(Camk2a-cre)T29-1Stl/J, Jackson Laboratories strain # 005359, MA, USA) were transfected bilaterally with AAV8-EF1α-FLEX-Chronos-GFP (5.1 * 10^12^ IU/ml) and 16 male littermates with AAV5-hSyn-FLEX-GFP (3.4 * 10^12^ IU/ml), targeting the vSUB using an angle of 25 (right hemisphere) or 23 (left hemisphere) degrees towards the midline at AP-3.3 mm, ML +/− 4.5 mm, and 3.3 mm distance from pia (Fig. 1a, left). Viral vector suspensions (80 nl) were infused (100 nl/min) through Hamilton syringes with bevelled needles (34 G), with the bevel facing laterally. The needle was left for 10 min before withdrawal to reduce backflow. Viral vectors were obtained from the University of North Carolina viral vector core. Optic fibres (1.25 mm ferrule diameter; Thorlabs, UK) cut to a length of 5.5–6 mm, were implanted bilaterally at AP −3.3 mm, ML +/− 2.8 mm from bregma and 3.9 mm below pia and fixed in place with dental cement and jewellers screws (Fig. 1a, right; Kemdent, UK). All experiments were in accordance to the Animal (Scientific Procedures) Act 1986, UK, and were approved by the Local Ethical Review Committee at the University of Oxford and the Home Office of the United Kingdom.

### Behavioural testing

#### Optogenetic stimulation

Green-light pulses were produced by a TTL-modulated DPSS laser (532 nm, CNI Lasers, China) using a computer-controlled pulse generator (Doric Lenses, Canada) and coupled to an optical fibre of 200 µm diameter and 0.39 NA (Thorlabs) via an adjustable collimator (CNI). Implanted optic fibres were connected via zirconia sleeves (Thorlabs) to an optic fibre cable attached via a commutator (Doric Lenses, Canada). For all optogenetic stimulations, 5 ms light pulses (power 2.0–5 mW, wavelength 532 nm) were delivered unilaterally at a frequency of 20 Hz for episodes of 5 s, alternating with 5 s pauses. Stimulation was given for either 1 min (Y-maze, ITI-phase), 2 min (Y-maze, test-phase), 3 min (all stimulations in the open field, Y-maze ITI + test-phase stimulation, elevated plus-maze) or 5 min (all remaining behavioural tests). Stimulation-sequences were delivered once per day, with a minimum 2 day recovery time for each hemisphere before subsequent stimulations. This was necessary as repeated stimulations with shorter intervals could increase the risk of inducing seizures in some animals. Animals experiencing optogenetically-induced seizures were not used in subsequent experiments and data from the experiment in which a seizure was observed were not included in any analyses. Optical power at the ferrule connected to the implanted optic fibre was measured before and after testing of each animal. Data for individual tests were excluded for Chronos-transfected animals if the power measured at the end of testing was more than 0.5 mW lower than the level determined during calibration (next section).

#### Optogenetically-induced hyperlocomotion

Mice of both groups were first optogenetically stimulated unilaterally for 3 min, starting at minute 15 of a 30 min exploration of an open field (l 43 cm, w 22 cm, h 20 cm; San Diego Instruments), and the number of infrared beam breaks was used as a locomotor activity readout. Starting at 2 mW, optical power was increased in 0.5 mW increments, and locomotor activity during optogenetic stimulation was measured; different hemispheres were tested on consecutive days. Calibration was terminated when hyperlocomotion of >115% of baseline (an average of the preceding 9 min baseline period) was observed twice at the same stimulation power in the same hemisphere in Chronos animals. The optimal power to be used in all subsequent experiments was determined for each animal individually from this calibration (LH: *n* = 7, RH: *n* = 11). GFP-transfected animals (controls) experienced a similar “calibration” alongside Chronos-transfected animals, to ensure comparable prior experience and control for non-specific effects of illumination. Power levels and the hemisphere receiving stimulation in control animals were matched to the Chronos group where possible. In cases where there were more GFP than Chronos animals, the power for the additional animals was set to intermediate values to ensure the same mean power in both groups. Unilateral stimulation, and the calibration of optical power for each animal was necessary to exclude animals displaying optogenetically-induced seizures, which we found were likely due to strong offsite expression in the dentate gyrus and/or CA3. To experimentally test the effects of optogenetic stimulation on locomotor activity, this procedure was repeated in a novel open field (40 × 40 cm, 25 cm wall height). Distance moved was measured in 3-minute bins via video-tracking (AnyMaze, San Diego Instruments, CA, USA). On a later occasion, the experiment was repeated twice in that same open field 1 h after injection of either 1 mg/kg of the D2-receptor antagonist raclopride (10 µl/g, i.p; Sigma-Aldrich, UK) or vehicle (saline; within-subject design). The order of drug exposure (raclopride/vehicle) was counterbalanced and there were three days between each test.

#### Effects of optogenetic stimulation on spatial novelty preference (SNP)

We assessed hippocampus-dependent short-term memory using the SNP Y-Maze task^45^. This task involves familiarizing animals with part of an environment, and then allowing exploration of both the familiar part of the environment and a novel area (see Fig. 2a). Due to rodents’ innate preference to explore novel spaces, animals should preferentially explore the novel area during the test phase^45,65^. For the SNP-test with optogenetic stimulation, mice were tethered to the optical fibre and left to acclimatize to a novel holding cage for 5 min before testing. They were then transferred to a transparent Y-shaped maze, filled with sawdust for 5 min, in which 1 of the 2 goal arms was blocked (sample phase). After a 1 min intra-trial interval (ITI) in the holding cage, mice were returned to the Y-maze for a 2 min test phase, in which both goal arms were accessible. Distance moved and time spent in each arm was measured via video-tracking (AnyMaze, San Diego Instruments, CA, USA). Mice were tested 4 times, in the same apparatus, but located in different rooms, and stimulation was applied during different stages of the task; either during (1) the sample phase (2) the ITI, (3) the test, or (4) throughout both the ITI and test phases (see Fig. 2a). To avoid the risk of overstimulation, sample-phase stimulation (5 min) was run last for all animals, and we did not stimulate throughout both the sample and test phases in the same trial. Locomotor data represent distance travelled in all available portions of the maze (i.e. all 3 arms in the test phase, familiar and start arm in the sample phase), unless stated otherwise.

#### Effects of amphetamine on SNP and locomotion

For pharmacological experiments, the same protocol was followed except that mice were injected with either amphetamine or saline-vehicle 30 min before testing (2.5 mg/kg, i.p; between-subject design) and placed in their home cage until 10 min before the sample phase when they were transferred to a novel holding cage (as above, Fig. 4a). A separate cohort of male wildtype mice was used (C57BL/6 J, Charles Rivers; *n* = 12 amphetamine treated, *n* = 11 vehicle).

#### Effects of optogenetic stimulation on anxiety in the elevated plus maze (EPM)

Animals were tested on an EPM (72 cm above ground) with two opposite grey closed arms (w 7 cm, l 38 cm, h 21 cm), and two open white arms (w 7 cm, l 38 cm). After tethering, mice had a 5 min acclimation period in a novel cage and optogenetic stimulation began in the last 20–30 s of this phase (see Fig. 3a). Mice were then placed in the centre of the EPM facing an open arm, and left to explore for 5 min, with optogenetic stimulation continuing for the first 2.5 min of testing (total stimulation time = 3 min). Distance moved was measured via video tracking (AnyMaze, San Diego Instruments, CA, USA), and time spent in each arm was scored manually offline, blind to experimental group. Data presented are from the first 2.5 min of the test (during optogenetic stimulation).

#### Effects of optogenetic stimulation on place preference (PP)

To test whether stimulation during the Y-Maze task was sufficient to induce a PP within the time-scale of minutes, we adapted the Y-Maze test to resemble a conditioned PP paradigm (see Fig. 3d). The start arm was blocked throughout, leaving accessible a V-shaped maze with two goal arms and a centre zone with removable doors for each arm. Animals were tethered throughout testing and were always introduced into the maze centre-zone with both doors closed. Animals went through two sample phases of 5 min, separated by a short ITI of 30–60 s, and received 5 min optogenetic stimulation during the second sample phase. In each sample phase only one arm was accessible (e.g. the left arm in the first phase and the right arm in the second phase, or *vice versa*). Animals were then placed in a holding cage for a 1 min ITI before being placed back into the maze. Both doors were removed, and mice were allowed to explore the entire maze for 2 min (test phase). Counterbalancing the order of stimulation was not possible given that the effects of stimulation on locomotion were found to last beyond the stimulation period in previous tests (see Fig. 1b,c), and might therefore carry-over to the time in the ‘non-stimulated’ arm given the short delays used here.

Animals were tested in a long-term PP task conducted in a modified 3–chamber apparatus (two 22 cm × 30 cm chambers joined by a 10 cm × 20 cm alley) over 3 days (see Fig. 3g). On day 1, animals were tethered (but not stimulated), placed in the central alley, and the sliding doors to both chambers were opened allowing exploration of the whole apparatus for 10 min. On day 2, animals began in the central alley and the door to one of the side compartments (left or right, counterbalanced) was opened. Once the animal entered the compartment the door was closed and the animal was left to explore for 5 min. For half the animals this first sample phase was paired with optogenetic stimulation. After a 2 h delay, animals were given a second sample phase (5 min) in the opposite chamber. Optogenetic stimulation was applied during the second sample phase for animals that did not receive it during the first sample. The following day, animals were placed in the central alley and both doors were opened to allow exploration of both chambers for 10 min. Animals were tethered for all phases of testing. Distance moved and time spent in each compartment were measured via video tracking (AnyMaze, San Diego Instruments, CA, USA). Only data for the first 5 min of the test phase are presented here.

### Histology

Animals were euthanized with sodium pentobarbital (Euthatal) and perfused transcardially with PBS followed by 4% paraformaldehyde (PFA). Head caps with optic fibres were detached carefully and the brains were removed and fixed in PFA and then transferred to phosphate buffered saline (PBS). Coronal slices (60 μm) were cut on a vibratome (Leica), washed in PBS, then PBS containing 4′,6-diamidino-2-phenylindole (DAPI, 1:1,000,000, Sigma) for 10 minutes. Slices were washed with PBS before mounting with Vectashield (Vector Labs, USA). Slices were scanned with a fluorescence microscope (Axio Zoom, Carl Zeiss) to detect DAPI and Chronos-GFP fluorescence.

### Analysis

Data were tested for normality using the Shapiro-Wilks Test. Where data for both groups was normally distributed, parametric tests (*t*-test or ANOVA) were applied, otherwise non-parametric equivalents were used. A 3-way repeated-measures ANOVA was used for the raclopride experiment despite data for some conditions not being normally distributed; however pairwise comparisons using appropriate non-parametric tests (not presented) yielded the same pattern of statistical significance. For *t*-test analyses, the adjusted *t*-values and df’s are reported for instances where Levene’s test for equality of variances indicated significant differences. In all cases, parametric and non-parametric tests yielded similar results.

## Data Availability

All data generated or analysed during this study are included in this article. All numeric source data for the presented figures are available from the corresponding author on reasonable request at the desired level of analysis.
